# Eliminating the AI digital divide by building local capacity

**DOI:** 10.1371/journal.pdig.0001026

**Published:** 2025-10-23

**Authors:** Freya Gulamali, Jee Young Kim, Kartik Pejavara, Ciera Thomas, Varoon Mathur, Zev Eigen, Mark Lifson, Manesh Patel, Keo Shaw, Danny Tobey, Alexandra Valladares, David Vidal, Jared Augenstein, Ashley Beecy, Sofi Bergkvist, Michael Burns, Michael Draugelis, Jesse M. Ehrenfeld, Patricia Henwood, Tonya Jagneaux, Morgan Jeffries, Christopher Khuory, Frank J. Liao, Vincent X. Liu, Chris Longhurst, Dominic Mack, Thomas M. Maddox, David McSwain, Steve Miff, Corey Miller, Sara G. Murray, Brian W. Patterson, Philip Payne, W. Nicholson Price II, Ram Rimal, Michael J. Sheppard, Karandeep Singh, Abdoul Sosseh, Jennifer Stoll, Corinne Stroum, Yasir Tarabichi, Sylvia Trujillo, Ladd Wiley, Alifia Hasan, Joan S. Kpodzro, Suresh Balu, Mark P. Sendak

**Affiliations:** 1 Duke Institute for Health Innovation, Durham, North Carolina, United States of America; 2 DLA Piper, United States of America; 3 Mayo Clinic, Rochester, Minnesota, United States of America; 4 Duke University Medical Center, Durham, North Carolina, United States of America; 5 Baker McKenzie, United States of America; 6 North Carolina Central University, Durham, North Carolina, United States of America; 7 Manatt Health, Los Angeles, California, United States of America; 8 NewYork-Presbyterian Hospital, New York, New York, United States of America; 9 Center for Care Innovations, Oakland, California, United States of America; 10 University of Michigan, Ann Arbor, Michigan, United States of America; 11 Geisinger Medical Center, Danville, Pennsylvania, United States of America; 12 Medical College of Wisconsin, Milwaulkee, Wisconsin, United States of America; 13 Jefferson Health, Philadelphia, Pennsylvania, United States of America; 14 Franciscan Missionaries of Our Lady Health System, Baton Rouge, Louisiana, United States of America; 15 American Medical Association, Chicago, Illinois, United States of America; 16 UW Health, Madison, Wisconsin, United States of America; 17 Kaiser Permanente Northern California, Oakland, California, United States of America; 18 UC San Diego Health, San Diego, California, United States of America; 19 Morehouse School of Medicine, Atlanta, Georgia, United States of America; 20 Washington University School of Medicine in St. Louis, St. Louis, Missouri, United States of America; 21 UNC Health, Chapel Hill, North Carolina, United States of America; 22 Parkland Center for Clinical Innovation, Dallas, Texas, United States of America; 23 Epic, Madison, Wisconsin, United States of America; 24 UCSF Health, San Francisco, California, United States of America; 25 Emory Health, Atlanta, Georgia, United States of America; 26 OCHIN, Portland, Oregon, United States of America; 27 SCAN Health Plan, Long Beach, California, United States of America; 28 MetroHealth, Cleveland, Ohio, United States of America; National Tsing-Hua University: National Tsing Hua University, TAIWAN

## Abstract

Over the past few years, health delivery organizations (HDOs) have been adopting and integrating AI tools, including clinical tools for tasks like predicting risk of inpatient mortality and operational tools for clinical documentation, scheduling and revenue cycle management, to fulfill the quintuple aim. The expertise and resources to do so is often concentrated in academic medical centers, leaving patients and providers in lower-resource settings unable to fully realize the benefits of AI tools. There is a growing divide in HDO ability to conduct AI product lifecycle management, due to a gap in resources and capabilities (e.g., technical expertise, funding, data infrastructure) to do so. In previous technological shifts in the United States including electronic health record and telehealth adoption, there were similar disparities in rates of adoption between higher and lower-resource settings. The government responded to these disparities successfully by creating centers of excellence to provide technical assistance to HDOs in rural and underserved communities. Similarly, a hub-and-spoke network, connecting HDOs with technical, regulatory, and legal support services from vendors, law firms, other HDOs with more AI capabilities, etc. can enable all settings to be well equipped to adopt AI tools. Health AI Partnership (HAIP) is a multi-stakeholder collaborative seeking to promote the safe and effective use of AI in healthcare. HAIP has launched a pilot program implementing a hub-and-spoke network, but targeted public investment is needed to enable capacity building nationwide. As more HDOs are striving to utilize AI tools to improve care delivery, federal and state governments should support the development of hub-and-spoke networks to promote widespread, meaningful adoption of AI across diverse settings. This effort requires coordination among all entities in the health AI ecosystem to ensure these tools are implemented safely and effectively and that all HDOs realize the benefits of these tools.

## Introduction

The emergence of generative AI offers new opportunities to advance the quintuple aim [1], which is defined by improving population health, enhancing the care experience, reducing costs, reducing burnout, and advancing health equity. Healthcare delivery organizations (HDOs) have seized opportunities to achieve these advancements at scale by investing in new partnerships to implement large language models (LLMs) that complement other AI tools.

Healthcare in the United States continues to suffer from low quality, low value care [2]. New investments in AI tools seek to help optimize the delivery of high-value interventions [3]. With record levels of physician burnout, ambient AI scribing solutions—AI tools that transcribe physician-patient interactions and summarize them into encounter notes—hold promise to help clinicians shift their focus from the documentation burden to enhancing patient interaction [4]. LLMs can also summarize information from discharge notes for patient accessibility [5] and retrieve information on patients’ health-related social needs from clinical notes to more effectively deploy resources to support patients [6]. Population health challenges are being addressed by other types of AI tools that can identify patients at risk for rapid disease progression [7] and expand patient access to psychotherapy [8].

Driven by urgency and competitive pressures to address healthcare challenges, many organizations are looking to adopt AI tools. According to one industry report, 76% of major payers and providers surveyed are seeking to establish AI pilots in the next year [9]. HDOs with the expertise and resources to implement administrative and clinical AI tools effectively are optimizing efficiency and improving quality of care. However, many HDOs are poorly equipped to conduct local AI quality management and are either not implementing AI tools at all or conducting limited testing and monitoring. The latter can exacerbate issues AI was designed to solve like improving efficiency and providing high quality care by increasing burden through alert fatigue, worsening inequities with biased predictions, negatively impacting care, and increasing liability on physicians and organizations. Implementing the same AI clinical decision support tool with workflows optimized for local context has been shown to reduce sepsis mortality in one context and be ineffective in another context [10,11]. Recent studies on the ROI of AI scribes are also showing mixed results across settings [12,13]. Substantial expertise is needed to realize the value of these tools, and ineffective AI implementations may negatively impact patients and clinicians while reinforcing existing biases [14] and discourage HDO uptake of beneficial technologies. While HDOs with resources and capabilities to leverage AI responsibly can achieve advancements in care, other HDOs may lag, driving a deeper digital divide and gap in quality of care.

### Digital divide in AI adoption

The digital divide is typically defined by lack of access to internet services, perpetuating socioeconomic disparities [15]. These disparities may be exacerbated as HDOs lacking personnel with relevant expertise, resources to purchase and localize tools, organizational processes and capabilities to conduct AI product lifecycle management, and IT infrastructure, including electronic health records (EHRs) will be unable to fully benefit from AI [16]. As of 2021, more than 20% of office-based practices did not adopt 2015 Certified EHR Technology, which serves as a backbone for many AI applications [17]. This disparity contributes to an AI divide, where HDOs with certified EHRs may have access to AI tools, while those without it are left at a disadvantage. The World Health Organization 2021 Guidance on AI Ethics and Governance in Health identifies the impact of the digital divide on AI adoption at a global scale and recognizes the potential for AI to improve health outcomes if greater efforts are made to address this divide [18].

The AI divide is particularly evident in one study, which found that only 61% of hospitals conduct local evaluations to assess for accuracy and 44% to assess for bias on most or all implemented AI products [19]. This finding suggests a gap in the resources or capabilities available to different HDOs to properly evaluate AI tools. Considering hospitals more commonly reported local evaluation on inpatient risk tools than outpatient administrative tools, authors of the study suggest one factor contributing to lower evaluation rates may be a misperception of the risk of outpatient tools. The AI divide, furthered by lack of awareness, resources, and capabilities to evaluate AI tools, can lead to unequal access to safe and effective AI use across different demographic subgroups, particularly among at-risk populations. As a result, there is a high risk of ineffective implementations that worsen inequities.

### Lessons from previous technological shifts in healthcare

Prior technological advancements that transformed healthcare delivery include the adoption and implementation of EHRs and telehealth. Both required federal support at the individual practice level, as well as incentives and infrastructure development to promote adoption. Federal programs that supported these transitions offer valuable examples for how the United States can rapidly facilitate the safe and effective adoption of AI tools.

The HITECH Act, enacted in 2009 and implemented in 2011, provided incentive payments to providers who adopted EHR systems. Of providers, 75% report that adopting EHR systems enabled them to deliver better patient care [20]. While these incentives drove adoption overall, small, non-teaching, and rural hospitals continued to lag behind [21]. To address this issue, the Office of the National Coordinator for Health IT (ASTP/ONC) established Regional Extension Centers (RECs) in 2010, aiming to increase EHR adoption in rural and underserved settings [22]. RECs were funded to deliver technical, legal, and financial assistance; education and training; assistance in vendor selection; privacy and security support; and more to help practices achieve meaningful use of EHRs. The SAFER guidelines checklist provided additional resources and information that stakeholders required, enabling practices to track their progress capturing value from EHRs [23]. By 2014, 89% of REC participants adopted all or part of EHRs compared to 58% of non-participants [24]. This demonstrated that financial incentives alone could not drive EHR adoption across all settings. Complementary professional services were essential to support smaller practices, and these services will be needed to promote responsible AI adoption across HDOs as well.

Similarly, Telehealth Centers for Excellence (COEs) and the National Consortium of Telehealth Resource Centers (TRCs) were created to provide professional support services, education, and training to facilitate digital health adoption in rural and underserved communities. The COEs, housed at the Medical University of South Carolina and University of Mississippi Medical Center, established telehealth-enabled locations nationwide and evaluated Telehealth Palliative Care and Tele-Behavioral Health programs [25].

The Health Resources and Services Administration (HRSA) provided $600,000 in the first year and $16.25 million over five years to support COEs. The 12 regional and two national TRCs, also funded by HRSA, played a critical role in advancing telehealth adoption during the COVID-19 pandemic [26]. These centers were complemented by infrastructure investments with the 2021 Bipartisan Infrastructure Law [27] and American Rescue Plan [28], which expanded access to broadband. Furthermore, the 2020 1135 Waiver [29] allowed Medicare to cover telehealth services more broadly. Philanthropies like the California Health Care Foundation complemented government funding to further catalyze adoption and evaluate program impact [30]. As a result, the percent of physicians whose practices had telehealth capabilities increased from 25.1% in 2018 to 74.4% in 2022 [31]. This growth in telehealth adoption also required a multi-pronged approach consisting of professional support services, infrastructure investments, and reimbursement changes.

In these major technological advancements in healthcare, capacity building programs played a crucial role in diffusing expertise to rural and underserved settings. They often took the form of a hub-and-spoke model, where RECs, COEs, and TRCs served as hubs, providing resources, professional services, and a peer learning community, while HDOs functioned as the spokes, receiving the support. Congress worked closely with federal agencies to empower HDOs to adopt EHR systems and telehealth through infrastructure investments, incentives, and hub-and-spoke networks. These EHR and telemedicine investments created significant value and ROI for a variety of stakeholders [32,33]. It is essential to draw insights from these examples, understanding how public and private entities partnered to promote EHR and telemedicine adoption, when addressing the current technological shift that is AI in healthcare. While the specific capabilities required and challenges involved in adopting AI differ from those posed by EHR systems and telehealth systems, the capacity building approach has proved valuable in past implementations and would be valuable to pursue in this case as well.

### Building capabilities before enforcing compliance

Currently, states are addressing the risks of AI in healthcare by strengthening protections related to patient privacy, consent, transparency, and non-discrimination (Table 1). At a federal level, there are protections, of which the degree of enforcement may alter with the change in administration (Table 2). The role of federal agencies is further complicated by the elimination of the Chevron deference, which previously had courts defer to federal agencies for interpretation of ambiguous statutes [34]. This could slow down enforcement of regulatory compliance at the federal agency level and put the onus on Congress to enact legislation in response to risks of AI. Agency efforts may shift towards providing support to enable safe and effective use of AI within HDOs.

**Table 1:** State regulatory landscape for AI in healthcare.

State	Law
Colorado	Colorado AI Act (2024): requirements on developers and deployers of high-risk AI systems for impact assessment and information disclosure.
California	California Consumer Privacy Act (2018): right to know how personal information is used, right to delete personal information, right to opt out of sale of personal information, and right to non-discrimination. Amended in 2020 with consumer right to correct inaccurate information and limit use. AB-3030 (2024): requires disclosure of generative AI use in patient communication. SB 1120 (2024): health insurer must ensure AI used for determinations are applied fairly and equitably. AB 2013 (2024): data used to train generative AI systems or services must be made apparent on developer websites.
Utah	Utah AI Policy Act (2024): requires disclosure of use of generative AI in advance of its use by regulated professionals, including physicians, and if prompted by a consumer in interactions regulated by Utah consumer privacy laws.
Texas	Texas Responsible AI Governance Act (pending): expected to establish obligations for developers, deployers, and distributors of high-risk AI systems.

**Table 2:** Federal regulatory landscape for AI in healthcare.

Federal Agency	Description of Resource
FDA	Clinical Decision Support Guidance Software as a Medical Device Good Machine Learning Practices
ASTP/ONC	HTI-1 final rule: algorithm transparency, interoperability, information blocking, etc. Health Information Technology Advisory Committee (HITAC)
OCR	Section 1557 of the Affordable Care Act: protections against discrimination with AI users legally responsible for managing risk. HIPAA Privacy and Security Rules: respond to complaints of violations.
FTC	Section 5(a) of the FTC Act: consumer protection from deceptive and unfair practices.
CMS	CPT Codes and NTAP enable billing for certain AI tools. FAQ Memo indicates Medicare Advantage organizations using AI cannot use these technologies to override standards for medical necessity in coverage determination
OSTP	AI Bill of Rights (rescinded): 5 principles for the design, use, and deployment of AI tools.

The patchwork of current regulatory efforts is mostly focused on one-size-fits-all compliance, where market access controls limit commercial availability of AI products and HDOs bear some burden to ensure that AI products are safe, effective, and equitable. This crude approach falsely assumes that all HDOs are equally equipped to bear this compliance burden and centers on the product rather than the numerous factors required for implementation. Similarly to how ONC established RECs to aid smaller, lower-resourced practices that were lagging in the adoption of EHR systems after the HITECH Act was passed, there must be accompanying investment in capacity building to help HDOs in achieving AI-related compliance requirements. In 2017, the disparity was still present as 93% of small, rural non-federal acute care hospitals had certified health IT compared to 99% of large hospitals [35]. If regulators and legislative bodies pursue a compliance-first approach that requires local validation of AI, low-resource HDOs will be unable to assess local AI performance and will be unable to utilize AI. On the other hand, if regulators and legislative bodies pursue a capacity building-first approach to support local validation of AI, low-resource HDOs will receive the necessary technical assistance to assess local AI performance and will be able to utilize AI. A compliance-first approach will widen the digital divide, whereas a capacity building-first approach can eliminate the digital divide.

This article advocates for local capacity building through a hub-and-spoke model for technical, operational, and educational assistance so that all HDOs can be empowered to realize the benefits of AI. Only after significant investment in capacity building and assistance establishing foundational AI capabilities can HDOs broadly bear complex compliance responsibilities.

## Hub-and-spoke model: Sharing AI lifecycle management processes

### Defining a hub-and-spoke model for capacity building

**Fig 1:**
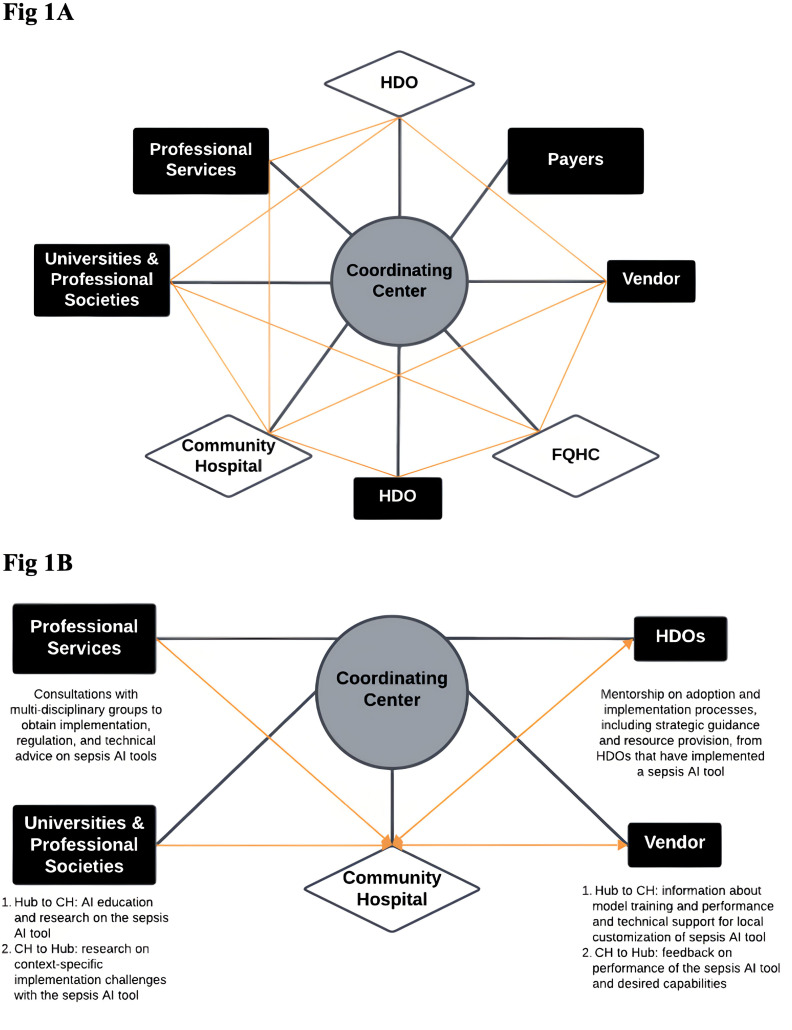
Hub-and-spoke network diagram for local capacity building. (A) The diagram above depicts a hub-and-spoke network where the coordinating center forms connections between hubs (professional services, payers, universities and professional societies, vendors, and other HDOs with more expertise in AI adoption and implementation) to spokes (community hospitals, federally qualified health centers, and other HDOs with less expertise in AI adoption and implementation). Black lines represent connection from hubs and spokes to the coordinating center, and orange lines represent connections from hubs to spokes facilitated by the coordinating center. Spokes may independently form partnerships with hubs not facilitated by the coordinating center, which is not illustrated in this diagram. (B) The diagram below demonstrates an example network for a community hospital seeking support in adopting and implementing a sepsis risk prediction tool. HDOs include university-affiliated medical centers as well as non-affiliated ones that have developed thorough expertise from having implemented a sepsis AI tool.

Capacity building requires engaging the appropriate stakeholders within an organization to build expertise and capabilities. This can occur through a hub-and-spoke model where hubs (which possess the necessary expertise and capabilities) provide training and support to spokes (that lack these attributes), enabling teams within spokes to implement AI tools within their organizations (Fig 1). Hubs can be specialized and interconnected to pool diverse expertise and balance workloads at a national level. The exchange of expertise would be bidirectional as spokes identify and raise awareness of challenges with adoption as well as unmet needs and share innovative approaches to conduct AI product lifecycle management (procurement, development, integration, and monitoring) in resource-limited environments. HAIP has detailed challenges and approaches to the AI product lifecycle, analyzing nearly 90 interviews to determine eight key decision points that HDOs experience when adopting and implementing an AI tool [16].

The hub-and-spoke approach, establishing resource centers that coordinate support services delivery to lower-resource HDOs, has succeeded in augmenting rates of EHR adoption after the HITECH Act was passed and advancing telehealth adoption, particularly during the pandemic. Applying this approach to AI capacity building can help address this new technological shift and reduce the AI divide by providing all HDOs with the specific set of support services they need to address the unique challenges of implementing AI tools safely and effectively.

### The network of hub stakeholders

Hubs have the potential to provide a range of technical and operational support services. They will require initial and ongoing funding to provide support services to external spoke sites, and such funding could be linked to the scope of services offered. Technical services could empower spoke sites to interface with vendors; identify the best solution within a product category; validate the performance of an AI solution locally; conduct AI risk assessments; and monitor an AI product post-implementation. Operational services could include supporting spokes to navigate ethical, legal, and regulatory challenges; develop and disseminate tools for program evaluation; and manage the AI product lifecycle. To enhance scalability, hubs can specialize in specific service areas (e.g., technical, regulatory, change management), specific AI use cases (e.g., sepsis care, chronic disease progression, mental health), or care delivery types (e.g., urban safety net, rural critical access hospital). Multiple hubs can collaborate to serve a spoke site, bringing together the necessary multidisciplinary capabilities and expertise.

Hubs may consist of HDOs that have invested significantly in developing AI capabilities as well as other expert stakeholders within the AI ecosystem to ensure broad adoption of AI in healthcare. Below, we describe potential relevant stakeholders.

#### Technological firms.

First, technology firms can play a critical role by providing tooling and infrastructure to integrate, evaluate, and continuously monitor AI products. AI product vendors should assume some of these responsibilities through service contracts, while cloud service providers can provide tooling to support these activities. Technology firms and universities are also spurring open-source innovation by releasing software that allows local evaluation of AI models with locally curated data for sensitivity, specificity, precision, and recall, publishing externally validated models, and partnering with HDOs to operationalize responsible AI principles [36–38]. Once AI tools are implemented, liability often lies on clinicians and local HDO facilities [39]. Both are often poorly equipped to safeguard against automation bias and inappropriate use of AI, especially as AI products increasingly use complex methods like neural networks and LLMs [40]. An HDO’s ability to make informed decisions when assessing safety and ROI also requires greater transparency from vendors. This includes the ability to assess bias, evaluate and monitor local product performance, and provide educational support to frontline users using tools like Model Fact Labels to enhance targeted clinical actions [41].

#### Payers.

Second, payers and states can identify AI use cases that are high value for HDOs and payers while funding assistance programs to support broad adoption. Payers like Blue Cross Blue Shield Michigan are providing incentives to HDOs using high-value AI tools [42]. These kinds of payer-driven programs that incentivize use of risk-stratification tools can improve patient care and reduce costly outcomes like readmission rates and ED visits. Coordination between payers to prioritize similar AI use cases with consistent performance measures can reduce the burden on HDOs procuring different tools for different payer programs. Payers with significant AI expertise may also be equipped to provide technical support services as a hub to spoke sites providing care to covered populations.

#### Professional service firms.

Third, professional service firms with implementation, regulatory, and data science expertise can provide site-specific support when working with proprietary and confidential data. These firms may provide a base set of services through a hub, with additional specialized services requiring further contracting.

#### Universities and professional societies.

Fourth, universities and professional societies can develop training programs to incorporate AI product lifecycle management skills into professional licensing and certification. Future generations of physicians, nurses, IT leaders, and healthcare administrators will need to understand how to manage AI systems integrated into care delivery.

#### Coordinating centers.

Hub sites would be a connected community to align recommendations to provide consistently high-quality support services across the nation. Objective measures of organizational maturity will be needed to identify potential hub sites that have invested thoroughly in AI capabilities and can provide AI technical and operational assistance. Ongoing monitoring and re-assessment of hub sites would ensure high-quality AI assistance to spoke sites. Significant effort will be required to coordinate diverse hub sites and ensure an equitable distribution of resources. This coordination will be facilitated by coordinating entities. Participation is beneficial for all stakeholders due to facilitation of contracts, increased ROI from utilizing AI tools, and reduced burden of adhering to varying standards.

### The network of spokes

Spokes might include community health centers, federally qualified health centers, community hospitals, Indian Health Service (IHS) facilities, VA Medical Centers (VAMCs), critical access hospitals, and other health systems with limited expertise in AI adoption. Ultimately, a set of freely accessible resources could be commissioned from experts within hub sites to benefit all HDOs. Organizational capabilities are dynamic and can evolve. Processes can be developed describing the steps a spoke site can take to transition to a hub site once they have sufficient internal capabilities and expertise. This train-the-trainer approach promotes sustainability and scalability of the hub-and-spoke model.

For spoke sites to benefit from hub services, each spoke could be best served by maintaining an in-house interdisciplinary team that includes clinical and operational staff (Table 3). This cross-functional team assumes responsibility for the quality and safety of AI implementations and collaborates with hubs to effectively evaluate and continuously monitor AI solutions within the local context. The ways in which members of this team come in during different points of implementation are described in Table 3. The core team must have a base set of capabilities, and this team can be composed of individuals both within the spoke site as well as individuals from professional service firms or hub organizations that complement the capabilities within the spoke site. AI product lifecycle management requires clinical and organizational leaders to gain additional expertise in generalized AI risk frameworks and set up feedback mechanisms for adverse event reporting to proactively ensure tools are working safely, effectively, and equitably. Spoke sites should have flexibility to pursue AI initiatives that align with their specific needs and organizational priorities. Long-term public sector financing to implement AI solutions and the accompanying IT infrastructure may be necessary to sustain this AI enablement for spoke sites.

**Table 3:** Core team within spoke sites recommended for AI adoption and implementation.

Expertise	Description
Project Management	Ensure milestone progression, coordinate stakeholder contributions
IT Team	Integration into EHR systems to automate data extraction, curation, and normalization, access data for evaluation, build secure and robust compute infrastructure
Analytics Team	Model evaluation to assess efficacy in operational workflows during, before, and after adoption, monitor for drift
Clinical Champion	Determine problem capability to meet clinical/operational needs, facilitate change management and buy-in from end-users
Senior Executive Sponsor	Assess priority of problem in context of organizational needs, dedicate resources and budget

### Coordinating entity for hub–spoke collaboration

Although a decentralized hub-and-spoke model enables dissemination of AI expertise across diverse HDOs, significant coordination efforts will be required to maximize benefit to spoke sites. A single spoke site aiming to implement a single AI product may need to draw upon expertise from multiple hub sites as well as receive support from other stakeholder groups listed above. Coordinating entities will be essential to assemble the capabilities and expertise needed to assist spoke sites through the various stages of the AI product lifecycle and disseminate lessons captured across settings. These coordinating centers can be established and funded by federal agencies (e.g., ONC grants funded RECs, HRSA grants fund TRCs) or state agencies (e.g., state Offices of Rural Health fund telehealth support within states). The agencies establishing these centers must also delineate the structure and authority of these centers. Day-to-day operations of these centers can vary but are likely to include meeting with spoke sites to identify needs while assessing the effectiveness of support services provided by hubs to address those needs.

### Implementing the hub-and-spoke model: Health AI Partnership Practice Network pilot program

A philanthropically funded demonstration project testing AI capacity building is already underway. Launched in 2024, the Health AI Partnership (HAIP) Practice Network program supports four FQHCs in southern California, Arizona, Texas, and Minnesota, and one community hospital in North Carolina in navigating the AI product lifecycle [43]. The program aims to build AI product lifecycle management capabilities within spoke sites and create a community of peer support and learning. Each HAIP Practice Network site has prioritized an internal use case, including an FDA-approved medical device, two generative AI scribe tools, and two EHR vendor-developed AI products. HAIP brings together capabilities across multiple hub sites, drawing on real-world AI expertise from HAIP members and contributors to host monthly Health AI Hubs on best practices for all spokes and hubs [44], an AI in Action Series for safety net organizations [45], and office hour sessions between hubs and spokes for more individualized supports.

Rather than imposing a compliance-first approach that may restrict innovation and AI use, the HAIP Practice Network supports HDOs in evaluating and integrating AI products prioritized by organizational needs. A rigorous independent evaluation of the program is being conducted so that learnings from this first demonstration project can inform future iterations of AI capacity building. There has been early news coverage of the pilot program detailing the program’s mission and early challenges [46,47]. The findings from the evaluation are not available at this time and will be shared in future work. These findings can shape the design of future hub-and-spoke networks, including the necessary degree and type of engagement from hubs, technical and personnel resources required at spokes, and the role of additional stakeholders.

While the program will generate valuable learnings by providing direct support to five HDOs, there is an urgent need for targeted public investment to scale the approach. There are 1,500 community health centers [48] and 6,200 hospitals [49] across the United States, many of which urgently need AI capacity building to optimize the safe and effective use of AI.

### Scaling lessons from the pilot program

In the next 12 months, federal and state agencies can design regional and use case-specific AI capacity building programs, issuing a call for proposals, and funding a portfolio of AI capacity-building demonstration projects. These projects should then be evaluated for improvements in efficiency and quality of care delivery to ensure that different stakeholders are delivering high-quality assistance services to support spoke sites in rural and underserved settings. Outcome measures for program evaluations must focus on scaling safe and effective use of AI rather than just rates of adoption. Not all AI solution integrations will be successful and AI capacity-building programs can increase investments in AI solutions that create value and eliminate investments in AI solutions that don’t. While public funding will promote sustainability of the hub-and-spoke network, philanthropy can play a significant role in designing and evaluating programs to demonstrate value as well as determining high-value-use cases of AI in various settings. Impact from philanthropy-funded programs can promote public investment and incentivize development of national programs. As one preliminary example, HAIP is working with The SCAN Foundation and California Health Care Foundation to develop a technical assistance program for California safety net providers. Similar programs should be designed, funded, implemented, and tested in other regions.

## Limitations

This article highlights the need for local capacity building to eliminate the AI digital divide through emphasizing the urgency of this issue and analyzing previous technological shifts. More information is necessary from pilot program learnings about how to most efficiently connect resources available in hubs to spokes to develop policies on how spokes can transition to hubs, the structure of coordinating entities, and other specific elements of the hub-and-spoke network. Rigorous evaluation of the HAIP Pilot Program is underway, and findings from this evaluation can provide further insight into how other hub-and-spoke networks should operate. The resources required by spokes and available from hubs differ significantly across settings. This means that designing and testing network structures is an essential next step to further understand the nuances in how these structures should differ across geographies, resource settings, and according to the AI use case.

## Conclusion

The digital divide between organizations that have the capacity to effectively utilize AI tools to enhance quality of care and address healthcare challenges and those that lack these capabilities is widening and calcifying. Similar to actions taken for EHR and telehealth technologies, there must be public and private investments in technical infrastructure and incentives to support widespread, meaningful adoption of AI across diverse HDO settings. In the past, the federal government invested in telehealth capabilities through HRSA and EHR adoption through ONC. State governments invested in health IT capabilities through state offices of rural health. Federal and state agencies have an opportunity to address the AI digital divide by supporting local capacity building, investing in infrastructure, and providing incentives for adoption. If coordination at the federal level proves politically infeasible, state-run programs piloting hub-and-spoke networks with financial incentives for AI adoption can also be an effective means to generate evidence of different approaches to support the safe and effective implementation of AI across diverse HDOs.

## Abbreviations:

HDOs: Healthcare delivery organizations
LLMs: large language models
EHRs: electronic health records
RECs: Regional Extension Centers
COEs: Centers for Excellence
TRCs: Telehealth Resource Centers
HRSA: Health Resources and Services Administration.
