# Correction: Eliminating the AI digital divide by building local capacity

**DOI:** 10.1371/journal.pdig.0001173

**Published:** 2025-12-30

**Authors:** Freya Gulamali, Jee Young Kim, Kartik Pejavara, Ciera Thomas, Varoon Mathur, Zev Eigen, Mark Lifson, Manesh Patel, Keo Shaw, Danny Tobey, Alexandra Valladares, David Vidal, Jared Augenstein, Ashley Beecy, Sofi Bergkvist, Michael Burns, Michael Draugelis, Jesse M. Ehrenfeld, Patricia Henwood, Tonya Jagneaux, Morgan Jeffries, Christopher Khoury, Frank J. Liao, Vincent X. Liu, Chris Longhurst, Dominic Mack, Thomas M. Maddox, David McSwain, Steve Miff, Corey Miller, Sara G. Murray, Brian W. Patterson, Philip Payne, W. Nicholson Price, Ram Rimal, Michael J. Sheppard, Karandeep Singh, Abdoul Sosseh, Jennifer Stoll, Corinne Stroum, Yasir Tarabichi, Sylvia Trujillo, Ladd Wiley, Alifia Hasan, Joan S. Kpodzro, Suresh Balu, Mark P. Sendak

The 22nd author’s name is spelled incorrectly. The correct name is: Christopher Khoury.

## References

[1] Gulamali F, Kim JY, Pejavara K, Thomas C, Mathur V, Eigen Z, et al. Eliminating the AI digital divide by building local capacity. PLOS Digit Health. 2025;4(10):e0001026. doi: 10.1371/journal.pdig.0001026 41129488 PMC12548875

